# Total Endovenous Laser Ablation Multicenter (TOTEM) trial: Early results

**DOI:** 10.1016/j.jvsv.2026.102554

**Published:** 2026-06-13

**Authors:** Luca Palombi, Jan Szczepański, Fabio Martinelli, Linas Velička

**Affiliations:** a Angiology and Advanced Surgical Phlebology Service, Villa Salus Foundation, Villa Salus Hospital, Venice Mestre, Italy; b Melius Clinic, Torun, Poland; c Clinic of Cardiac, Thoracic and Vascular Surgery, Medical Academy, Lithuanian University of Health Sciences, Kaunas, Lithuania

**Keywords:** TEVLA, EVLA, Varicose veins, Endovenous ablation, Chronic venous disease

## Abstract

**Objective:**

To evaluate the safety and efficacy of total endovenous laser ablation (TEVLA) in a multicenter cohort.

**Methods:**

This prospective multicenter nonrandomized study included 153 patients (Clinical, Etiologic, Anatomic, Pathophysiologic Classification [CEAP] C2–C6). TEVLA was performed as a single-session procedure for the treatment of truncal veins, tributaries, and perforators. The primary end point was the occlusion rate. The secondary end points included complications, pain (visual analog scale [VAS]), and revised Venous Clinical Severity Score (rVCSS) improvement.

**Results:**

The occlusion rate was 99.3% at 7 days and 98.3% at 6 months. No deep vein thrombosis or endothermal heat-induced thrombosis events were observed. Minor complications included hyperpigmentation (5.9%) and paresthesia (2.6%). The mean rVCSS significantly improved (−3.24 at 1 month; *P* < .001). No association was found between linear endovenous energy density and complications.

**Conclusions:**

TEVLA is a safe and effective single-step technique with high occlusion rates and low complication rates. Further randomized studies are warranted.

**Article Highlights**

• **Type of Research:** Prospective multicenter nonrandomized clinical study
• **Key Findings:** In a cohort of 153 limbs treated with total endovenous laser ablation (TEVLA), the occlusion rate was 99.3% at 7 days and 98.3% at 6 months, with no cases of deep vein thrombosis or endothermal heat-induced thrombosis. Complication rates were low, and significant improvement was observed in the revised Venous Clinical Severity Score.
• **Take Home Message:** TEVLA represents a safe and effective single-session strategy for the comprehensive treatment of truncal reflux and varicose tributaries, potentially reducing the need for adjunctive procedures.

Chronic venous disease (CVD) is a highly prevalent condition associated with significant morbidity and a substantial burden on health care systems worldwide. Over the last two decades, endovenous thermal ablation (EVTA) has progressively replaced conventional surgery as the first-line treatment for saphenous vein incompetence, owing to its high occlusion rates, reduced postoperative recovery time, and improved patient-reported outcomes.^1^^,^^2^

Despite the well-established efficacy of truncal ablation, the optimal management of varicose tributaries remains under debate. The current clinical guidelines support different strategies, including concomitant or staged treatment with ambulatory phlebectomy or ultrasound-guided foam sclerotherapy (UGFS), without reaching a clear consensus on the most effective approach.^3^^,^^4^ In this context, several studies have suggested that untreated incompetent tributaries may contribute to higher recurrence and reintervention rates, potentially affecting long-term outcomes.^5^^,^^6^

Conversely, in previous studies, combined approaches led to a reduction in reinterventions and improvements in early disease severity, and quality of life scores were better in the concomitant group, although these approaches may increase procedural complexity and raise concerns regarding complications.^7^^,^^8^

Total endovenous laser ablation (TEVLA) has been introduced as a single-step technique designed to treat truncal veins, tributaries, and perforators simultaneously. This approach aims to simplify treatment strategies and reduce the need for adjunctive procedures. However, concerns persist regarding its safety profile, its reproducibility, and the potential risk of adverse events, particularly when treating superficial tributaries.

Evidence supporting TEVLA remains limited, especially in multicenter prospective settings. The Total Endovenous Laser Ablation Multicenter (TOTEM) trial was therefore designed to evaluate the feasibility, safety, and early effectiveness of a comprehensive single-session endovenous treatment strategy for truncal veins, tributaries, and perforators, rather than to reassess the established efficacy of conventional truncal endovenous laser ablation (EVLA) alone across different institutions. The primary objective of this study was to assess the occlusion rate following TEVLA, and the secondary objectives included the evaluation of procedural parameters, complication rates, pain perception, and clinical improvement.

## Methods

The TOTEM trial was designed as a prospective, multicenter, nonrandomized, nonblinded clinical study conducted across three European vascular centers, namely Hospital Villa Salus in Venice (Italy), Melius Clinic in Toruń (Poland), and Kaunas Medical University Hospital in Kaunas (Lithuania). This study was conducted in accordance with the principles of the Declaration of Helsinki, after obtaining approval from the institutional review board (ethics committee), and all participants provided written informed consent before enrolment.

A total of 153 consecutive patients affected by symptomatic CVD were included in this study. All patients were classified within CEAP clinical classes C2–C6 and presented with reflux involving truncal veins, including the great saphenous vein (GSV), small saphenous vein (SSV), or accessory saphenous veins (ASVs), in association with incompetent tributaries and/or perforating veins. Patients with CEAP C1 disease were excluded, as were those with acute deep vein thrombosis (DVT), significant peripheral arterial disease defined by an ankle-brachial index below 0.8, active malignancy or recent oncologic treatment within the previous 5 years, and severe systemic comorbidities involving the major organs. Additional exclusion criteria were lower limb infections such as cellulitis, pregnancy or breastfeeding, ongoing treatment with systemic steroids or hemostatic agents, and any condition considered unsuitable for patient participation by the investigators.

The following baseline variables were assessed: sex, age, thrombophilia, previous superficial venous thrombosis (SVT), previous DVT, obesity, revised Venous Clinical Severity Score (rVCSS), CEAP clinical class, laterality (right vs left limb), treated truncal segment (GSV, SSV, or anterior ASV [recorded in the database as ASV]), maximum target vein diameter, diameter measured 2 cm from the saphenofemoral or saphenopopliteal junction, treated length, number of 16-gauge needle cannulas, and total tumescence volume.

All enrolled patients underwent TEVLA as a single-session procedure. The primary efficacy end point of this study was the occlusion rate of the treated veins, which was assessed using duplex ultrasound study at predefined follow-up intervals, including 7 days, 1 month, and 6 months, with additional longer-term follow-up planned.

The secondary efficacy end points included the evaluation of procedural parameters, specifically power and linear endovenous energy density (LEED), and the need for adjunctive treatments or reinterventions.

Safety end points were assessed through the evaluation of perioperative and postoperative complications, including endothermal heat-induced thrombosis (EHIT), DVT, superficial vein thrombosis, bleeding events, paresthesia, and hyperpigmentation. Pain perception was measured using a visual analog scale (VAS). Clinical improvement was further assessed using the rVCSS at baseline and during follow-up visits.

Demographic, clinical, and procedural data were extracted from the study database of the multicenter TEVLA cohort. Because each database row corresponded to a treated limb/procedure, descriptive analyses were performed at the limb/procedure level. Records with analyzable baseline demographic and procedural information were included in the present analysis.

### Laser equipment

As study equipment, we used laser control units manufactured by neoLaser Ltd. (Neov1940) and cylindric monoring fiber (Infinite Fiber, NeoLaser “CORONA Infinite Ring Fiber,” Light Guide Optics International Ltd.). The laser fiber incorporates a fully fused radial-emitting tip and it is available in two configurations: 600 *μ*m core fiber with an external diameter of 1800 *μ*m and a 400 *μ*m fiber with an external diameter of 1300 *μ*m. The tip provides homogeneous 360° radial laser emission over a 4 mm segment through a multiple concentric emission rings.

### Statistical analysis

Statistical analysis was performed in accordance with contemporary reporting standards for interventional venous studies. Continuous variables are presented as mean ± standard deviation (SD) or median with interquartile range (IQR), as appropriate based on the data distribution, whereas categorical variables are reported as absolute counts and percentages. The primary end point was treated as a binary outcome and summarized as proportions with corresponding 95% confidence intervals (CIs), which were calculated using both the exact Clopper–Pearson method and the Wilson method to ensure the robustness of the estimation.

Comparative inference for the primary end point was conducted using a one-sample exact binomial test against a prespecified performance threshold. The secondary end points involving paired measurements (eg, rVCSS changes over time) were analyzed using the Wilcoxon signed-rank test given the non-normal distribution of differences. Time-to-event data for vein occlusion durability were evaluated using Kaplan-Meier survival analysis, with the results reported as estimated probabilities with 95% CIs.

Given the extremely low number of failure events, multivariable modeling using mixed-effects logistic regression was deemed statistically unreliable and was therefore not performed. All statistical tests were two-sided, with the significance level set at *P* < .05. Analyses were conducted using standard statistical software packages commonly adopted in clinical research (IBM SPSS Statistics v.26 and Microsoft Excel, v. 2021).

### Procedure description

Each procedure was preceded by the written acquisition of accurate informed consent from the patient. Surgery was conducted as outpatient treatment. On the day of surgery, a preoperative ultrasound study was performed to exclude DVT and to obtain hemodynamic mapping, with the patient in the standing position. The following three types of ultrasound machines were used: SonoScape P80 Elite (SonoScape Medical Corp), Philips iU22 ultrasound system (Philips Healthcare), and Philips EPIQ Elite 8 Cardio ultrasound system (Philips Healthcare).

The procedures were conducted with percutaneous access, using 6F or 4F introducers for main access and 16 G needles at the operator's discretion and according to the size and numbers of the collateral vessels to be treated. The entire procedure was performed under ultrasound guidance. The interventions were conducted with the administration of only local anesthesia by tumescence (500 mL of saline solution cooled at 4 °C with two vials of 10 mL of lidocaine, 20 mg/mL), delivered using a peristaltic pump under ultrasound guidance. In cases of a markedly incompetent terminal valve, as determined by the operator, flush ablation was performed by positioning the laser fiber in close proximity to the terminal valve. In all other cases of saphenous trunk ablation, the procedure was performed according to the standard technique, in which ablation was initiated 2 cm distal to the junction. All procedures were completed with the treatment of the collateral varicose veins through EVLA. The laser thermoablation procedure was performed with the pullback technique. Energy delivery was not determined solely based on the vessel diameter. Although the target vessel caliber represented an important parameter, the final ablation settings were individualized according to multiple procedural and anatomical factors, including the treated venous segment, depth from the skin surface, vessel tortuosity, quality of tumescence, tactile feedback during fiber withdrawal, and real-time ultrasound assessment of vein wall response and closure. Accordingly, LEED selection was adapted intraoperatively based on both anatomical characteristics and dynamic ultrasound-guided procedural findings. Tumescent local anesthesia solution was infiltrated around the veins. Tumescence, in addition to protecting the surrounding tissues (primarily the skin), has the fundamental purpose of increasing the contact of the laser fiber with the venous wall and creating hydrodissection. The patient was placed in the Trendelenburg position to empty the veins. Then, the 400-μm fiber was inserted into the lumen of each vein, and laser ablation was then performed. At the end of surgery, an ultrasonographic closure check was performed, and elastic sock monocollant class II was applied.

## Results

### Baseline demographic, clinical, and anatomical characteristics

A total of 153 treated limbs/procedures were available for descriptive analysis (Table I). Women accounted for 98 cases (64.1%) and men for 55 (35.9%). The mean age was 52.7 ± 15.7 years, with a median age of 55 years (IQR, 41–63 years). Regarding baseline clinical characteristics, thrombophilia was not recorded in any case (0%). A history of SVT was present in 15 patients (9.8%), whereas previous DVT was reported in 1 patient (0.65%). Obesity was documented in 33 patients (21.5%). The mean rVCSS was 7.7 ± 2.7, with a median value of 7 (IQR, 6–9). The clinical distribution of CEAP was weighted toward intermediate clinical stages. Specifically, C2 was observed in 38 cases (24.8%), C3 in 78 (50.9%), C4 in 31 (20.2%), C5 in 5 (3.2%), and C6 in 3 (1.9%); no limb was classified as C1.

**Table I:** Baseline demographic, clinical, and procedural characteristics

Variable	Value
Number of treated limbs/procedures	158
Age, mean ± SD, years	52.7 ± 15.7
Age, median (IQR)	55 (41–63)
Female sex, n (%)	76 (64.4)
Male sex, n (%)	42 (35.6)
Thrombophilia, n (%)	0 (0)
History of SVT, n (%)	15 (12.7)
History of DVT, n (%)	1 (0.8)
Obesity, n (%)	33 (28.0)
rVCSS, mean ± SD	7.7 ± 2.7
rVCSS, median (IQR)	7 (6–9)
C2	38 (24.8)
C3	78 (50.9)
C4	31 (20.2)
C5	5 (3.2)
C6	3 (1.9)
Right limb	73 (47.7)
Left limb	80 (52.3)
GSV	127 (83.0)
AASV/ASV	26 (16.9)
SSV	21 (13.7)
Maximum vein diameter, mean ± SD, mm	10.6 ± 4.8
Maximum vein diameter, median (IQR)	9.0 (7.5–12.0)
Diameter at 2 cm from the junction, mean ± SD, mm	8.5 ± 3.1
Diameter at 2 cm from junction, median (IQR)	8.0 (6.0–10.0)
Treated length, mean ± SD, cm	43.6 ± 17.9
Treated length, median (IQR)	45.0 (32.0–55.0)
Number of 16G needle cannulas, mean ± SD	3.4 ± 3.6
Number of 16G needle cannulas, median (IQR)	3.0 (1.0–5.0)
Total tumescence, mean ± SD, mL	342.1 ± 260.4
Total tumescence, median (IQR)	300 (125–500)

*AASV*, Anterior accessory saphenous vein; *ASV*, accessory saphenous veins; *DVT*, deep vein thrombosis; *GSV*, great saphenous vein; *IQR*, interquartile range; *rVCSS*, revised Venous Clinical Severity Score; *SD*, standard deviation; *SSV*, small saphenous vein; *SVT*, superficial venous thrombosis.

Percentages for treated truncal segments are not mutually exclusive, as multiple segments could be treated in the same procedure.

Laterality was balanced, with 73 right limbs (47.7%) and 80 left limbs (52.3%). The most commonly treated truncal segment was GSV, which was involved in 127 procedures (83.0%). Anterior ASV/ASV treatment was recorded in 26 procedures (16.9%) and SSV treatment in 21 (13.7%). Because some procedures involved more than one truncal segment, these percentages are not mutually exclusive.

From an anatomical perspective, the mean maximum vein diameter was 10.6 ± 4.8 mm, with a median value of 9.0 mm (IQR, 7.5–12.0 mm). The mean diameter measured 2 cm from the junction was 8.5 ± 3.1 mm, with a median value of 8.0 mm (IQR, 6.0–10.0 mm). The mean treated length was 43.6 ± 17.9 cm, with a median value of 45.0 cm (IQR, 32.0–55.0 cm).

Procedurally, the mean number of 16-gauge needle cannulas was 3.4 ± 3.6, with a median of 3.0 (IQR, 1.0–5.0). The mean total tumescence volume was 342.1 ± 260.4 mL, with a median value of 300.0 mL (IQR, 125.0–500.0 mL).

### Primary end point

**Fig 1:**
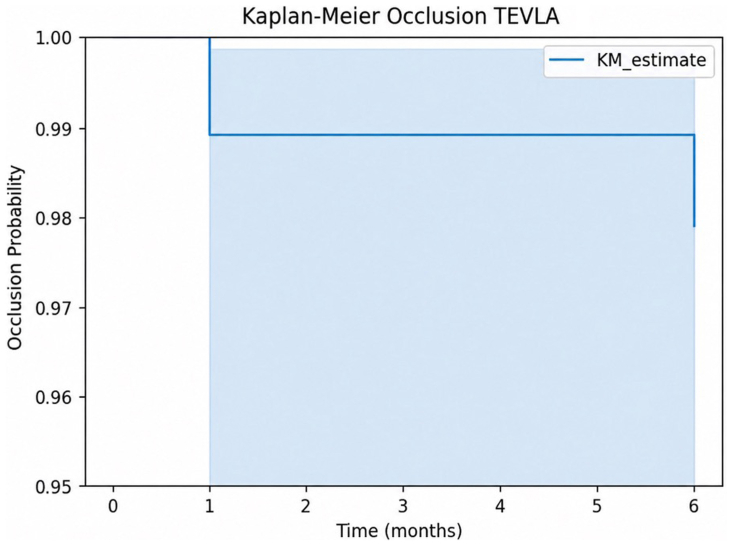
Kaplan-Meier curve demonstrating the probability of target vein occlusion following total endovenous laser ablation (*TEVLA*) over a 6-month follow-up period. The occlusion rate was 100% at baseline and remained high, with a slight decline to approximately 98% to 99% at 6 months. The shaded areas indicate 95% confidence intervals.

At the 7-day and 1-month follow-up points, vein occlusion was achieved in 152 of 153 patients, corresponding to an occlusion rate of 99.35%, and the occlusion rate at 6 months was 98.3%. The exact 95% Clopper–Pearson CI was 96.41%–99.98%, and the Wilson 95% CI was 96.39%–99.88%. Due to the occurrence of only one occlusion failure, multivariable mixed-effects logistic regression was not considered reliable and was therefore not performed (Fig 1).

### Energy parameters

**Fig 2:**
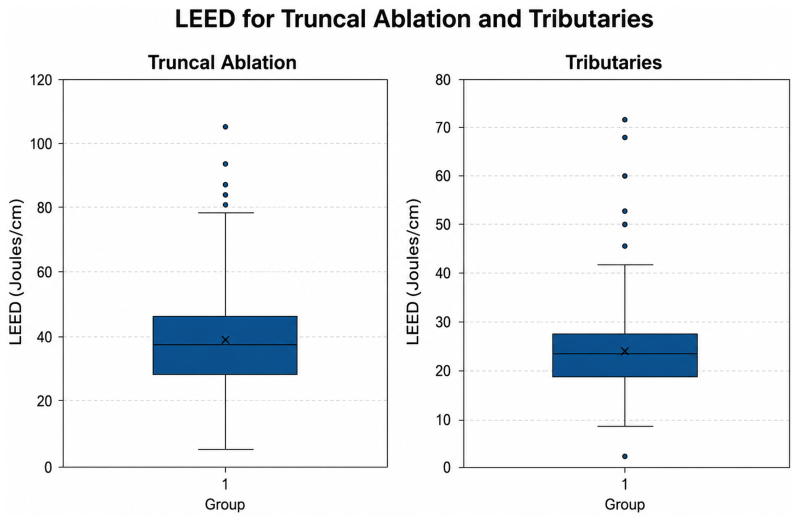
Box-and-whisker plots (box plots) of linear endovenous energy density (*LEED*) (J/cm) applied during truncal vein ablation procedures (*left panel*; great saphenous vein, small saphenous vein, and accessory saphenous vein) and tributary treatment (*right panel*). For truncal ablation, the median LEED was approximately 35–40 J/cm, with an interquartile range (IQR) of about 28–48 J/cm. For tributary treatment, the median LEED was lower at approximately 24 to 26 J/cm, with an IQR of about 20 to 29 J/cm. In both groups, only a limited number of higher-energy outliers were observed. Overall, the distributions suggest a standardized and controlled application of LEED, with lower energy levels generally used for tributary treatment and limited variability across procedures.

For truncal ablation, 174 treated truncal segments were available for analysis. The mean power output was 5.02 ± 0.95 W, with a median value of 5.0 W (IQR, 4.0–6.0 W). The mean LEED was 45.32 ± 14.16 J/cm, with a median value of 44.0 J/cm (IQR, 39.0–48.9 J/cm) (Fig 2). For tributary ablation, 153 procedures were analyzed. The mean power output was 3.83 ± 1.51 W, with a median value of 4.0 W (IQR, 2.0–5.0 W), whereas the mean LEED was 26.50 ± 9.88 J/cm, with a median value of 25.5 J/cm (IQR, 20.0–29.0 J/cm) (Fig 2).

### Secondary end points

No case of EHIT (class 1–4) or DVT was observed (0%). Superficial vein thrombosis occurred in one patient (0.7%). Minor complications included paresthesia in four patients (2.6%) and hyperpigmentation in nine patients (5.9%). No bleeding events were reported.

Postprocedural pain at the first follow-up point was low, with a mean VAS score of 1.39 ± 1.06 and a median score of 1 (IQR, 1–2; range, 0–5).

**Fig 3:**
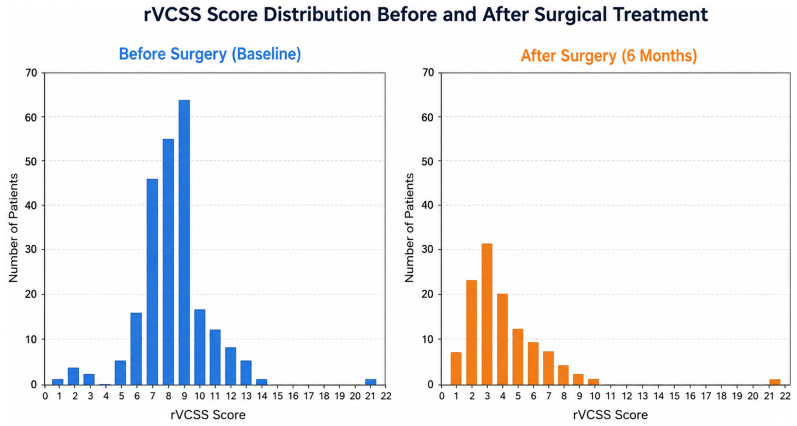
Distribution of revised Venous Clinical Severity Score (*rVCSS*) values in patients before surgery and at the 6-month follow-up. A clear leftward shift of the distribution is observed after treatment, indicating a reduction in rVCSS scores and, consequently, an overall clinical improvement. Before surgery, rVCSS values are predominantly clustered in the moderate-to-high range, whereas after surgery, the distribution shifts toward lower scores, with a higher concentration of patients in the mild disease range. This pattern reflects a consistent improvement in venous clinical severity across the cohort following surgical intervention. *rVCSS*, revised Venous Clinical Severity Score.

The mean baseline rVCSS was 7.95 ± 2.56, decreasing to 4.71 ± 2.36 at the 1-month follow-up point. This corresponded to a mean change of −3.24 points and a median change of −3 (IQR, −4 to −2), with a statistically significant improvement on Wilcoxon signed-rank testing (*P* < .001). Among 57 patients with 6-month follow-up, the mean rVCSS decreased from 8.53 ± 1.92 at baseline to 5.04 ± 2.59, with a mean change of −3.49 points and a median change of −3 (IQR, −4 to −2), again showing significant improvement (*P* < .001) (Fig 3).

## Discussion

The present multicenter trial provides further evidence supporting the feasibility, efficacy, and safety of TEVLA as a single-step strategy for the treatment of truncal reflux and associated varicose tributaries. The main findings of this study can be summarized as follows: (1) a very high early occlusion rate for TEVLA comparable to that of standard endovenous thermal techniques; (2) a low incidence of complications; and (3) the absence of a statistically significant association between higher delivered energy (LEED) and the occurrence of hyperpigmentation or paresthesia.

The observed occlusion rates of 99.3% at 7 days and 98.3% at 6 months are consistent with the outcomes reported in randomized controlled trials comparing EVLA with surgical stripping, where occlusion rates typically exceed 90% to 95% in the short term.^1^^,^^2^ Although no direct comparative arm was included in the present study, the early occlusion rate observed after TEVLA appears consistent with the published outcomes of standard truncal endovenous thermal ablation, including EVLA and RFA. These results confirm that extending endovenous treatment to tributaries and perforators within a TEVLA approach does not compromise the efficacy of truncal ablation.

A central concern regarding TEVLA has been its potential to increase the incidence of complications due to the more extensive treatment field, particularly when superficial tributaries are targeted. In this context, the analysis of safety end points is particularly relevant. In the present study, hyperpigmentation occurred in 5.9% and paresthesia in 2.6% of patients, rates that are low and clinically acceptable.

Importantly, no statistically significant association was observed between LEED values and the occurrence of these complications (hyperpigmentation: *P* = .499; paresthesia: *P* = .433) (Table II). This result suggests that within the energy ranges used in this study, higher energy delivery does not translate into increased tissue damage or neural injury. This result is clinically relevant, as it challenges the common perception that more aggressive energy settings may inherently increase complication rates, and it instead supports the concept that procedural technique, tumescence, and fiber technology may play more critical roles.^9^ From a pathophysiological perspective, the lack of a correlation between LEED and complications may be explained by several procedural factors. The use of tumescent anesthesia provides a protective thermal buffer, reduces the vein diameter, and increases the distance between the treated vein and surrounding tissues, thereby limiting collateral damage. In addition, the use of radial or ring fibers ensures a more homogeneous distribution of thermal energy, potentially reducing focal overheating and the risk of skin or nerve injury.

**Table II:** Association between delivered energy (LEED) and occurrence of adverse events

Outcome	Group	N	Mean LEED (J/cm)	SD	*P* value
Hyperpigmentation	Yes	10	27.56	10.14	.499
	No	143	26.44	9.90	
Paresthesia	Yes	5	29.00	9.09	.433
	No	148	26.43	9.92	

*LEED*, Linear endovenous energy density; *SD*, standard deviation.

This table summarizes the relationship between the mean LEED values (J/cm) and the occurrence of two clinical outcomes (hyperpigmentation and paresthesia) observed during the trial. For each outcome, the mean LEED values were compared between patients who developed the event (“yes”) and those who did not (“no”).

No statistically significant differences were found in the mean LEED values between the groups for either hyperpigmentation or paresthesia.

*P* values derived from comparison between the groups (event vs no event).

When contextualizing these findings within the literature, the rates of paresthesia observed in the TOTEM trial appear lower than those reported for ambulatory phlebectomy. According to the Society for Vascular Surgery and American Venous Forum (SVS-AVF) guidelines, paresthesia rates following phlebectomy range widely, from 9.5% to up to 39%, depending on the technique and follow-up duration.^3^ In contrast, paresthesia rates after EVLA only of truncal veins are typically reported to be around 3%, which is consistent with the findings of the present study.^3^^,^^4^

Similarly, the European Society for Vascular Surgery (ESVS) guidelines report hyperpigmentation rates of approximately 4.6% following phlebectomy and up to 10%–15% after UGFS.^4^ In this context, the rate of 5.9% observed in the TOTEM trial falls within the lower range of reported values for minimally invasive treatments and appears comparable, if not favorable, when contrasted with UGFS.

Notably, the absence of an increased complication rate for TEVLA compared with that of UGFS is particularly relevant, given that TEVLA replaces the need for sclerosant injection and avoids its associated risks, including allergic reactions and neurological events. Previous randomized and comparative studies have shown that hyperpigmentation after UGFS may occur in up to 30% of cases, although the rates vary depending on the concentration and technique.^4^^,^^5^ The findings of the present study therefore suggest that TEVLA provides a comparable safety profile while simplifying treatment into a single-session procedure.

Another important aspect is the comprehensive nature of TEVLA. As highlighted in previous systematic reviews and meta-analyses, untreated tributaries are associated with higher rates of recurrence and reintervention.^7^^,^^8^ By addressing all incompetent venous segments in a single session, TEVLA may reduce the need for secondary procedures without increasing complication rates, as is also suggested by comparative data showing similar safety profiles between concomitant and staged treatments.

The clinical improvement observed in rVCSS further supports the effectiveness of this approach, with significant reductions in rVCSS already evident at 1 month and sustained at 6 months. These findings align with those of previous studies demonstrating rapid symptom relief following endovenous interventions.^1^^,^^2^^,^^10^

This study has several limitations that should be acknowledged. First, the nonrandomized design limits the ability to directly compare TEVLA with other treatment modalities such as UGFS or phlebectomy. Second, the relatively short follow-up currently available for a subset of patients precludes definitive conclusions regarding long-term durability and late complications. Third, the low number of adverse events limits the statistical power for detecting subtle associations between procedural variables and complications.

Nevertheless, the strengths of this study include its prospective multicenter design, standardized data collection, and detailed analysis of procedural parameters, including energy delivery. The consistency of findings across different centers supports the reproducibility of the TEVLA technique.

## Conclusions

The preliminary results of the TOTEM trial suggest that TEVLA is a safe and effective approach for the comprehensive treatment of CVD. The absence of a significant association between higher LEED and the occurrence of hyperpigmentation or paresthesia, together with complication rates comparable to or lower than those reported for established techniques such as UGFS and phlebectomy, supports the adoption of this strategy in appropriately selected patients. Further randomized studies with longer follow-up are warranted to confirm these findings and to better define the role of TEVLA within the current treatment algorithms. TEVLA should be considered a promising comprehensive single-session strategy with favorable early safety and effectiveness, but not as a definitive replacement for established approaches until randomized long-term comparative data are available.

## Author Contribution

Conception and design: LP

Analysis and interpretation: LP, FM

Data collection: LP, JS, FM, LV

Writing the article: LP

Critical revision of the article: LP, JS, FM, LV

Final approval of the article: LP, JS, FM, LV

Statistical analysis: LP

Obtained funding: Not applicable

Overall responsibility: LP

## Ethics Statement

The study was conducted according to the Declaration of Helsinki. Ethical approval was obtained and all patients provided informed consent.

## Funding

None.

## Disclosures

None.
